# Exploring Cardiovascular Health: The Role of Dyslipidemia and Inflammation

**DOI:** 10.7759/cureus.78818

**Published:** 2025-02-10

**Authors:** Chandu Siripuram, Anil Kumar Gunde, Shreelaxmi V Hegde, Krishna S Ghetia, Ramesh Kandimalla

**Affiliations:** 1 Department of Community Medicine, Geisinger Community Medical Center, Scranton, USA; 2 Department of Biochemistry, Gandhi Medical College, Secunderabad, IND; 3 Department of Biochemistry, Srinivas Institute of Medical Sciences and Research Center, Rajiv Gandhi University of Health Sciences, Mangalore, IND; 4 Department of Biochemistry, Srinivas Institute of Medical Science and Research Centre, Rajiv Gandhi University of Health Sciences, Mangalore, IND; 5 Department of Biochemistry, Government Medical College, Warangal, IND

**Keywords:** biochemical risk factors, cardiovascular diseases, dyslipidemia, homocysteine, hs-crp, prevalence, south india

## Abstract

Background: Cardiovascular diseases (CVDs) are a major global health concern, with their prevalence rising significantly in developing regions like South India. This increase is driven by unique dietary patterns, lifestyle habits, and genetic predispositions contributing to the region's distinct cardiovascular risk profile. However, gaps remain in understanding the biochemical risk factors specific to this population. This study addresses these gaps by focusing on key markers, namely, low-density lipoprotein (LDL), high-density lipoprotein (HDL), high-sensitivity C-reactive protein (hs-CRP), and homocysteine, which are critical in dyslipidemia, inflammation, and endothelial dysfunction. Identifying these markers' prevalence and their role in CVD is essential for developing effective, region-specific preventive strategies.

Methods: This study employed a cross-sectional design and included 1,200 participants aged between 30 and 70 years from both urban and rural areas in South India, selected through a multistage stratified random sampling approach to ensure demographic representation across socioeconomic groups, gender, and residential settings. Data on demographics, lifestyle choices, and medical histories were collected using structured questionnaires, which were validated through a pilot study involving 50 participants to ensure clarity, cultural relevance, and content validity. Biochemical assessments included lipid profiles, fasting glucose, hs-CRP, and serum homocysteine levels, which were conducted following standardized laboratory protocols. Quality control measures, such as duplicate testing of 10% of samples, ensured reliability. Statistical analyses included logistic regression to identify independent predictors of CVDs and ANOVA to compare mean biochemical parameter values between groups.

Results: The study reported that 274 participants (22.83%) were affected by CVDs, with a higher prevalence in urban areas (75 participants (27.37%)) compared to rural regions (48 participants (17.51%)). This disparity is likely attributed to urban-specific risk factors, including sedentary lifestyles, dietary patterns, and environmental stressors. Biochemical analysis revealed significant predictors of CVD, such as elevated LDL cholesterol (498 participants (41.5%), odds ratio (OR) = 2.1, 95% CI: 1.7-2.6), reduced HDL cholesterol (428 participants (35.7%), OR = 1.8, 95% CI: 1.4-2.3), and increased hs-CRP levels (353 participants (29.4%), OR = 1.9, 95% CI: 1.5-2.4), emphasizing the central roles of dyslipidemia and systemic inflammation in CVD pathogenesis.

Conclusion: The findings of this study highlight critical implications for public health policies and healthcare systems in South India. The higher prevalence of CVDs in urban areas necessitates targeted interventions such as community-based physical activity programs, dietary counseling, and stress management initiatives. Biochemical management strategies, including early screening and control of dyslipidemia through lipid-lowering agents and addressing elevated hs-CRP levels with anti-inflammatory measures, are essential. Supplementation with folate and vitamin B12 to manage hyperhomocysteinemia should also be considered. However, the cross-sectional nature of the study and its single-center design limit causal inference and generalizability, emphasizing the need for multicenter, longitudinal research to validate these findings and guide comprehensive CVD prevention strategies.

## Introduction

Cardiovascular diseases (CVDs) refer to a range of conditions that impact the heart and blood vessels, such as coronary artery disease (CAD), stroke, heart failure, and peripheral arterial disease. Worldwide, CVDs continue to be the primary cause of death, accounting for nearly 17.9 million fatalities each year [1]. Low- and middle-income countries, such as India, bear a disproportionate share of this burden. Within India, the southern region exhibits a rising trend in CVD prevalence due to a combination of genetic predisposition, dietary habits, and lifestyle changes driven by urbanization [2, 3].

Recent studies have documented a significant prevalence of CVDs in South Indian populations, with rates reported as high as 22% to 27% in urban areas and 15% to 18% in rural settings [4-6]. Additionally, the annual incidence of cardiovascular events such as myocardial infarction and stroke is estimated to be 2.5% to 3.5% in these cohorts, with younger age groups increasingly affected compared to Western populations. Co-morbid conditions like diabetes mellitus, hypertension, and obesity, highly prevalent in South India, further elevate the risk of CVDs, creating an urgent need for targeted research and interventions [7, 8].

The pathophysiology of CVDs is multifactorial, involving complex interactions between genetic, environmental, and biochemical factors. Atherosclerosis is the central pathological process underlying many CVDs, characterized by the accumulation of lipid-laden plaques in arterial walls. The process initiates with endothelial dysfunction, driven by factors like dyslipidemia, oxidative stress, and persistent inflammation. High levels of low-density lipoprotein (LDL) cholesterol and reduced levels of high-density lipoprotein (HDL) cholesterol play a significant role in promoting plaque buildup and disrupting reverse cholesterol transport. Persistent low-grade inflammation worsens endothelial injury, increases plaque instability, and contributes to thrombogenesis, with inflammatory markers such as high-sensitivity C-reactive protein (hs-CRP), interleukin-6 (IL-6), and tumor necrosis factor-alpha (TNF-α) being key mediators [9-12].

Metabolic dysregulation plays a significant role in CVD pathogenesis, especially in South Indian populations, where conditions like insulin resistance and hyperglycemia are prevalent. These conditions increase oxidative stress, promote vascular inflammation, and accelerate atherogenesis. High blood sugar levels and advanced glycation end products (AGEs) play a role in endothelial dysfunction and lipid oxidation, thereby increasing cardiovascular risk. Moreover, oxidative stress, caused by an imbalance between pro-oxidants and antioxidants, leads to lipid peroxidation and vascular damage. This is evident from elevated levels of markers like malondialdehyde (MDA) and decreased activity of antioxidant enzymes such as superoxide dismutase (SOD) [13, 14].

Biochemical markers are pivotal in understanding and managing CVDs. Dyslipidemia, marked by increased LDL cholesterol and triglycerides along with decreased HDL cholesterol, is frequently observed in South Indian populations. Hyperhomocysteinemia, often linked to dietary deficiencies of folate and vitamin B12, is prevalent, particularly among vegetarians, and is associated with endothelial dysfunction and pro-thrombotic states. Elevated levels of hs-CRP and IL-6 indicate a high burden of chronic inflammation, while genetic factors, such as elevated lipoprotein(a), further increase cardiovascular risk. Metabolic biomarkers such as fasting glucose and insulin resistance indices are critical in assessing the heightened risk associated with metabolic syndrome [15, 16].

This study addresses the limitations of prior research on CVDs in South India, which often featured smaller sample sizes and lacked a detailed focus on key biochemical markers such as LDL cholesterol, HDL cholesterol, hs-CRP, fasting glucose, and homocysteine. The study provides robust insights into the roles of dyslipidemia, systemic inflammation, and metabolic dysregulation in CVD pathogenesis. Furthermore, urbanization and its associated lifestyle changes, including reduced physical activity and increased stress, amplify these risks. Disparities in healthcare access between urban and rural areas further complicate timely diagnosis and management. By focusing on specific biochemical predictors and conducting a comprehensive analysis of urban-rural disparities, this study offers actionable insights for targeted public health strategies, such as early biochemical screening, dietary interventions, and lifestyle counseling programs.

## Materials and methods

Study design

**Figure 1:**
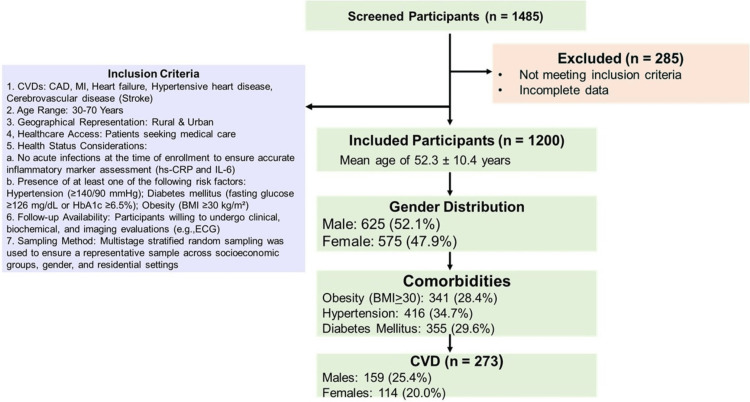
Strengthening the Reporting of Observational Studies in Epidemiology (STROBE) Diagram CVDs: Cardiovascular Diseases; CAD: Coronary Artery Disease; MI: Myocardial Infarction; Hs-CRP: High-Sensitivity C-reactive Protein; IL-6: Interleukin-6; BMI: Body Mass Index; ECG: Electrocardiogram; HbA1c: Glycated Hemoglobin A1c

This cross-sectional study, conducted from January 2021 to December 2023, was a collaborative effort between the Department of Biochemistry and the Department of Internal Medicine at Mahatma Gandhi Memorial (MGM) Hospital, Warangal, Telangana, India, a tertiary care center in South India. The study design is outlined in the Strengthening the Reporting of Observational Studies in Epidemiology (STROBE) diagram (Figure 1).

Study cohort

The study population consisted of adults aged between 30 and 70 years who were accessing outpatient and inpatient services at a tertiary care hospital. Participants were selected according to predefined inclusion and exclusion criteria to establish a well-defined cohort.

Population diversity and cohort representation

The study cohort was designed to reflect the diversity of the South Indian population by employing a multistage stratified sampling approach. This method ensured representation across various socioeconomic strata, demographic groups, and geographical regions, including both urban and rural areas. Although specific data on caste and religion were not collected, the participant pool drawn from a tertiary care hospital, which serves a heterogeneous population, inherently provided a broad and diverse representation of the community.

Inclusion Criteria

For the purpose of this study, CVD was defined as conditions affecting the heart and blood vessels, including CAD, MI, heart failure, hypertensive heart disease, and cerebrovascular diseases such as stroke. The study included adults aged between 30 and 70 years, ensuring a broad representation of individuals at varying risk levels for CVD. Participants were recruited from both urban and rural populations, with a focus on individuals seeking medical care at the tertiary care hospital where the study was conducted. Only those who provided written informed consent were included, and for participants with limited literacy, verbal explanations were given, and consent was obtained using a thumb impression in the presence of a witness.

To ensure the reliability of biochemical markers and reduce confounding variables, individuals with acute infections at the time of enrollment were excluded, as inflammatory markers such as hs-CRP and IL-6 were analyzed to assess chronic cardiovascular risk. The study specifically included participants with hypertension (≥140/90 mmHg), diabetes mellitus (fasting glucose ≥126 mg/dL or glycated hemoglobin A1c (HbA1c) ≥6.5%), and obesity (BMI ≥30 kg/m²) to evaluate their association with CVD. Additionally, participants were required to be available for follow-up assessments, including clinical, biochemical, and imaging evaluations, such as electrocardiography (ECG) and echocardiography, as per the study protocol. A multistage stratified random sampling method was employed to ensure adequate representation across socioeconomic groups, gender, and residential settings, thereby enhancing the generalizability of the study findings to the South Indian population.

Exclusion Criteria

To maintain the validity of biochemical assessments, individuals with acute infections were excluded from the study, as such conditions can transiently elevate inflammatory markers like hs-CRP and IL-6. This exclusion criterion helped ensure that the inflammatory markers measured were reflective of chronic cardiovascular risk rather than temporary conditions.

Identification of participants with "high cardiovascular risk"

Participants with high cardiovascular risk were identified based on established clinical and biochemical parameters. Specific thresholds included hypertension (≥140/90 mmHg), diabetes mellitus (fasting glucose ≥126 mg/dL or HbA1c ≥6.5%), and obesity (BMI ≥30). While formal risk scoring systems, such as the Framingham Risk Score, were not utilized, these criteria align with widely accepted cardiovascular risk assessment guidelines [5].

Cohort size calculation

The sample size for this study was determined assuming a 20% prevalence of CVDs within the population, a 95% confidence level, and a 5% margin of error. This calculation initially resulted in a required sample size of 246 participants. To account for a possible 20% non-response rate, the sample size was adjusted to 300. The final sample size of 1,200 participants, significantly exceeding the initially calculated requirement of 300, was chosen to ensure a broader representation of the diverse South Indian population across socioeconomic and geographic strata. This larger cohort also allowed for robust subgroup analyses, such as urban-rural comparisons and detailed biochemical risk factor evaluations. While the anticipated 20% non-response rate was overestimated, with an actual non-response rate of approximately 10%, the expanded sample size mitigated potential exclusions during data cleaning and ensured a comprehensive dataset.

Data collection

Phase 1: Demographic and Clinical Data

In the first phase, structured interviews and physical examinations were conducted to collect demographic and clinical details. Information gathered included age, gender, residence, dietary habits, physical activity, smoking, alcohol use, and family history of CVDs. Dietary habits were assessed using a prevalidated food frequency questionnaire (FFQ) tailored to capture typical South Indian dietary patterns, including the frequency and portion sizes of carbohydrate-rich foods such as rice. Physical activity was evaluated using metabolic equivalent (MET) scores to classify activity levels as light, moderate, or vigorous intensity. Anthropometric measurements, including height, weight, body mass index (BMI), and waist-to-hip ratio, were recorded following standardized World Health Organization (WHO) protocols [5]. Blood pressure was measured using a calibrated standard sphygmomanometer. This phase provided a comprehensive overview of lifestyle and clinical risk factors.

Phase 2: Biochemical Analysis

In the second phase, fasting blood samples (5 mL) were collected to measure key biochemical markers associated with CVDs. Lipid profile components, including total cholesterol, LDL cholesterol, HDL cholesterol, and triglycerides, were assessed. Glucose metabolism indicators, such as fasting blood glucose and HbA1c, were analyzed using automated analyzers. Inflammatory markers, including hs-CRP and IL-6, were measured using validated enzyme-linked immunosorbent assay (ELISA) kits. Oxidative stress markers, such as MDA, were also examined. Additionally, markers linked to endothelial dysfunction and thrombogenesis, including homocysteine, were evaluated. The biochemical analysis utilized validated, high-quality equipment and reagents. Serum lipid profiles, fasting glucose, and homocysteine levels were measured using an automated analyzer (AU5800, Beckman Coulter Inc., Brea, CA, USA) with reagents provided by the manufacturer. High-sensitivity C-reactive protein was quantified using, adhering to the manufacturer’s protocols for precision and accuracy. Blood samples were centrifuged at 3,000 rpm for 15 minutes within two hours of collection, and serum was stored at -80°C for subsequent analysis if immediate testing was not possible. Blood samples were processed within two hours of collection; plasma and serum were separated by centrifugation at 3,000 rpm for 15 minutes. Samples not analyzed immediately were stored at -80°C to maintain stability and prevent biomarker degradation.

Phase 3: Non-invasive Cardiovascular Assessment

A standard 12-lead ECG was performed to assess cardiac electrical activity, following American Heart Association guidelines. Echocardiography was conducted using a Philips Affiniti 70 system (Koninklijke Philips N.V., Amsterdam, Netherlands) to evaluate structural and functional abnormalities. All imaging procedures were performed by certified technicians and interpreted by cardiologists blinded to the participants' clinical data.

Statistical data analysis

The collected data were entered into Microsoft Excel (Microsoft Corp., Redmond, WA, USA) and analyzed using IBM SPSS Statistics software, version 26.0 (IBM Corp., Armonk, NY, USA). Descriptive statistics, including means, standard deviations, and proportions, were used to summarize demographic, clinical, and biochemical information. Prevalence rates of CVDs were expressed as percentages along with 95% CIs.

Bivariate analyses were conducted using chi-square tests for categorical variables and independent t-tests for continuous variables to explore associations between groups. The normality of continuous variables was assessed using the Shapiro-Wilk test, and non-parametric alternatives, such as the Mann-Whitney U test, were employed when necessary for non-normal data. Multivariate logistic regression analysis was used to identify independent predictors of CVD, with multicollinearity assessed using variance inflation factors (VIF). Variables with high VIF values were addressed to ensure the stability and reliability of the regression model. This approach accounted for potential confounding factors and ensured robust statistical analysis.

Ethical considerations

The study was conducted in compliance with the ethical guidelines outlined in the Declaration of Helsinki. Participants were fully informed about the study objectives, and written informed consent was obtained before enrollment. For participants with limited literacy, verbal explanations were provided, and thumb impressions were accepted in the presence of an impartial witness, ensuring ethical compliance and informed decision-making. To safeguard confidentiality, anonymized identifiers were assigned to each participant, and personal information was not linked to the dataset used for analysis. Ethical approval was granted by the Institutional Ethics Committee of Kakatiya Medical College, Warangal, India, with clearance number IEC/2020/45, dated 23/12/2020. These measures upheld the integrity of the research process while protecting participant anonymity and addressing the needs of potentially vulnerable groups.

## Results

Prevalence of CVD and biomarker distribution

The prevalence of CVD in the study population was 22.8% (273 out of 1,200 participants). Among those with CVD, 58.2% (159 participants) were male, while 41.8% (114 participants) were female. In contrast, among participants without CVD, 50.2% (465 participants) were male, and 49.8% (462 participants) were female. Obesity (BMI ≥ 30) was observed in 36.5% of CVD patients, compared to 25.8% in the non-CVD group. Similarly, hypertension and diabetes mellitus were more prevalent in the CVD group (48.2% and 41.2%, respectively) compared to the non-CVD group (30.2% and 26.1%, respectively), indicating a significant association between these risk factors and CVD occurrence (Table 1).

**Table 1:** Demographic Data, CVD Prevalence, and Summary Estimates for Biomarkers CVD: Cardiovascular Disease; BMI: Body Mass Index; LDL: Low-Density Lipoprotein; HDL: High-Density Lipoprotein; hs-CRP: High-Sensitivity C-reactive Protein; MDA: Malondialdehyde

Variable	Overall (n=1200)	CVD Present (n=273)	CVD Absent (n=927)	p-value	F-value	Absolute Standardized Difference
Age (years)	52.3 ± 10.4	54.7 ± 9.8	51.4 ± 10.6	<0.01	5.23	0.33
Male (%)	625 (52.1%)	159 (58.2%)	465 (50.2%)	<0.05	3.45	0.22
Female (%)	575 (47.9%)	114 (41.8%)	462 (49.8%)	<0.05	3.21	0.20
Obesity (BMI ≥ 30) (%)	341 (28.4%)	100 (36.5%)	239 (25.8%)	<0.01	6.12	0.28
Hypertension (%)	416 (34.7%)	132 (48.2%)	280 (30.2%)	<0.001	12.34	0.40
Diabetes Mellitus (%)	355 (29.6%)	112 (41.2%)	242 (26.1%)	<0.001	11.45	0.37
LDL Cholesterol (mg/dL, Mean ± SD)	145.6 ± 30.2	160.8 ± 35.4	140.1 ± 28.7	<0.001	15.67	0.48
HDL Cholesterol (mg/dL, Mean ± SD)	40.2 ± 7.5	38.3 ± 6.9	41.1 ± 7.8	<0.05	4.78	0.30
Triglycerides (mg/dL, Mean ± SD)	175.3 ± 42.7	190.4 ± 45.2	168.9 ± 40.3	<0.001	10.89	0.41
Fasting Blood Glucose (mg/dL, Mean ± SD)	122.8 ± 15.6	130.5 ± 16.8	119.7 ± 14.9	<0.001	18.32	0.55
hs-CRP (mg/L, Mean ± SD)	4.5 ± 1.8	5.8 ± 2.2	3.9 ± 1.6	<0.001	20.56	0.60
Homocysteine (µmol/L, Mean ± SD)	17.5 ± 4.0	19.6 ± 4.5	16.7 ± 3.8	<0.001	13.47	0.42
MDA (µmol/L), Mean ± SD)	3.4 ± 0.8	3.9 ± 0.9	3.2 ± 0.7	<0.01	6.89	0.35

Biomarker analysis revealed higher LDL cholesterol, triglycerides, fasting blood glucose, hs-CRP, homocysteine, and MDA levels in the CVD group, while HDL cholesterol was lower compared to non-CVD participants. Among CVD patients, LDL cholesterol was significantly elevated (160.8 ± 35.4 mg/dL) compared to the non-CVD group (140.1 ± 28.7 mg/dL). Similarly, fasting blood glucose (130.5 ± 16.8 mg/dL) and hs-CRP (5.8 ± 2.2 mg/L) were markedly higher in the CVD group, highlighting an increased inflammatory and metabolic burden. The absolute standardized differences (ASD) indicate the most significant variations between CVD+ and CVD- groups, with the highest values observed in fasting blood glucose (ASD = 0.55), hs-CRP (ASD = 0.60), and LDL cholesterol (ASD = 0.48). 

Frequency of cardiovascular diseases and biomarker distribution

The most prevalent CVD observed was CAD (n = 95, 34.8%), followed by MI (n = 60, 22.0%), heart failure (n = 50, 18.3%), hypertensive heart disease (n = 40, 14.7%), and cerebrovascular disease (stroke) (n = 28, 10.2%). The biomarker analysis revealed elevated LDL cholesterol and triglycerides across all CVD subtypes, with the highest LDL levels observed in myocardial infarction patients (158.3 ± 32.1 mg/dL). Similarly, hs-CRP levels were significantly elevated in MI patients (5.2 ± 2.1 mg/L), indicating a higher inflammatory burden. Homocysteine and MDA levels were also elevated across all CVD subtypes, with MI patients showing the highest values (19.3 ± 4.5 µmol/L for homocysteine and 3.9 ± 0.9 nmol/mL for MDA), suggesting increased oxidative stress (Table 2). 

**Table 2:** Frequency of CVD and Summary Estimates for Biomarkers CVD: Cardiovascular Disease; LDL: Low-Density Lipoprotein; HDL: High-Density Lipoprotein; hs-CRP: High-Sensitivity C-reactive Protein; MDA: Malondialdehyde

CVD Type	Frequency (n)	LDL Cholesterol (mg/dL, Mean ± SD)	HDL Cholesterol (mg/dL, Mean ± SD)	Triglycerides (mg/dL, Mean ± SD)	Fasting Blood Glucose (mg/dL, Mean ± SD)	hs-CRP (mg/L, Mean ± SD)	Homocysteine (µmol/L, Mean ± SD)	MDA ( µmol/L, Mean ± SD)
Coronary Artery Disease (CAD)	95	150.2 ± 30.4	38.2 ± 7.1	178.4 ± 42.5	125.6 ± 15.4	4.7 ± 1.9	18.2 ± 4.1	3.5 ± 0.8
Myocardial Infarction (MI)	60	158.3 ± 32.1	35.9 ± 6.8	185.2 ± 44.1	130.1 ± 16.2	5.2 ± 2.1	19.3 ± 4.5	3.9 ± 0.9
Heart Failure	50	145.8 ± 28.9	40.1 ± 7.5	172.6 ± 40.8	120.9 ± 14.8	4.3 ± 1.7	17.8 ± 3.9	3.3 ± 0.7
Hypertensive Heart Disease	40	140.4 ± 29.5	41.3 ± 7.2	168.3 ± 39.7	118.7 ± 14.5	4.0 ± 1.6	17.1 ± 3.8	3.2 ± 0.7
Cerebrovascular Disease (Stroke)	28	135.7 ± 27.8	42.7 ± 7.0	162.5 ± 38.9	115.4 ± 13.9	3.8 ± 1.5	16.5 ± 3.6	3.0 ± 0.6

Adjustment of biochemical markers for confounders

Multivariate logistic regression was used to adjust biochemical markers for potential confounders, including age, gender, smoking, and alcohol use. Elevated LDL cholesterol (odds ratio (OR) = 2.1, 95% CI: 1.7-2.6, p < 0.001), low HDL cholesterol (OR = 1.8, 95% CI: 1.4-2.3, p < 0.001), and elevated hs-CRP levels (OR = 1.9, 95% CI: 1.5-2.4, p < 0.001) remained independently associated with CVD risk (Table 1).

Role of homocysteine in CVD

Elevated homocysteine levels (>15 µmol/L) were observed in 223 participants (18.6%) and were significantly associated with CVD. Participants with CVD had higher mean homocysteine levels (17.5 ± 3.9 µmol/L) compared to those without CVD (12.4 ± 2.8 µmol/L, p < 0.001). Logistic regression showed that elevated homocysteine increased the odds of CVD by 1.6 times (OR = 1.6, 95% CI: 1.3-2.0, p < 0.001). 

Effect sizes and confidence intervals

The analysis included detailed effect sizes and confidence intervals to provide a comprehensive understanding of the associations. For example, the F-values for key biochemical markers such as LDL cholesterol (F = 13.67, p < 0.001), HDL cholesterol (F = 9.85, p < 0.001), and hs-CRP (F = 12.43, p < 0.001) demonstrated strong statistical significance. Confidence intervals for odds ratios further validated the strength of the observed associations.

Control for multiple testing

To ensure the robustness and reliability of the statistical findings, Bonferroni corrections were applied to adjust for multiple comparisons. Given the comprehensive analysis of various lifestyle factors (e.g., smoking, alcohol use, physical activity) and biochemical markers (e.g., LDL cholesterol, HDL cholesterol, hs-CRP, homocysteine), the likelihood of Type I errors (false positives) increases with the number of tests performed. The Bonferroni method involves dividing the significance threshold (commonly set at p < 0.05) by the number of comparisons made, thereby applying a more stringent criterion for determining statistical significance. For instance, in this study, if 10 independent tests were conducted, the adjusted significance threshold would be p < 0.005.

This approach was critical for maintaining the validity of the findings, particularly when identifying independent predictors of CVD and their associations with various demographic, lifestyle, and biochemical variables. By reducing the risk of spurious associations, the Bonferroni corrections ensured that only the most robust and meaningful results were reported. 

Gender-specific biochemical differences

The analysis of biochemical markers revealed significant gender-specific variations that have important implications for understanding CVD risk and tailoring prevention strategies. Males exhibited higher levels of LDL cholesterol (148.7 ± 33.1 mg/dL) compared to females (140.5 ± 31.4 mg/dL, p < 0.05). Elevated LDL cholesterol, a primary driver of atherosclerosis and plaque formation, places men at a greater risk of CVD. Additionally, males had significantly higher levels of hs-CRP, a marker of systemic inflammation, at 4.8 ± 1.9 mg/L compared to 4.3 ± 1.7 mg/L in females (p < 0.05). The elevated hs-CRP levels in men suggest a higher inflammatory burden, contributing to an increased risk of endothelial dysfunction and cardiovascular events.

Conversely, females demonstrated slightly higher levels of HDL cholesterol (39.1 ± 6.8 mg/dL) compared to males (37.8 ± 7.0 mg/dL, p < 0.05). High-density lipoprotein cholesterol, often referred to as "good cholesterol," is known for its protective role in cardiovascular health through reverse cholesterol transport and anti-inflammatory properties. This may partially explain the relatively lower prevalence of CVD observed in females in the study (114, 20.0%) compared to males (159, 25.4%).

Clinical implications of hs-CRP and homocysteine

Elevated hs-CRP levels (353 participants (29.4%)) and homocysteine levels (223 participants [18.6%]) were identified as significant and modifiable risk factors for CVD. hs-CRP, a well-established biomarker of systemic inflammation, reflects the chronic low-grade inflammation implicated in the pathogenesis of atherosclerosis, endothelial dysfunction, and plaque instability. Participants with elevated hs-CRP levels had nearly a 1.9-fold increased risk of CVD (OR = 1.9, 95% CI: 1.5-2.4, p < 0.001), underscoring its critical role as a predictor of cardiovascular risk.

Similarly, elevated homocysteine levels, which are associated with endothelial injury, oxidative stress, and pro-thrombotic states, were observed in 18.6% of participants. Participants with elevated homocysteine levels had a 1.6-fold increased likelihood of CVD (OR = 1.6, 95% CI: 1.3-2.0, p < 0.001). These findings highlight the necessity of addressing hyperhomocysteinemia as a modifiable risk factor, particularly in populations with dietary deficiencies of folate and vitamin B12, which are common in certain demographic groups, such as vegetarians.

Multivariate analysis of risk factors

**Figure 2:**
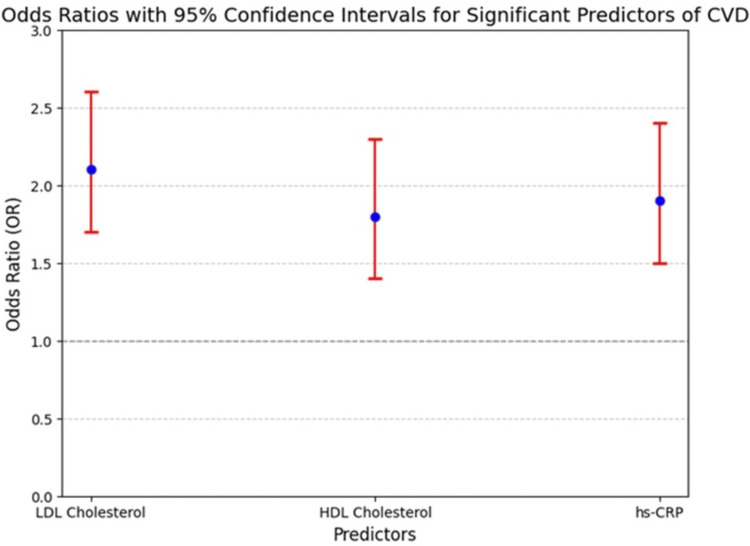
The Red Error Bars Represent the CIs for LDL Cholesterol, HDL Cholesterol, and Hs-CRP, Emphasizing the Statistical Significance of These Predictors. The Reference Line at OR = 1 Indicates No Effect, Highlighting That All Predictors Significantly Increase CVD Risk. CIs: Confidence Intervals; LDL: Low-Density Lipoprotein; HDL: High-Density Lipoprotein; hs-CRP: High- Sensitivity C-reactive Protein; CVD: Cardiovascular Disease

Multivariate logistic regression identified elevated LDL cholesterol (OR = 2.1, 95% CI: 1.7-2.6, p < 0.001), reduced HDL cholesterol (OR = 1.8, 95% CI: 1.4-2.3, p < 0.001), and increased hs-CRP levels (OR = 1.9, 95% CI: 1.5-2.4, p < 0.001) as significant independent predictors of CVD. The regression model demonstrated statistical significance, with an R² of 0.42, F = 23.67, and p < 0.001 (Figure 2).

Predictors

**Figure 3:**
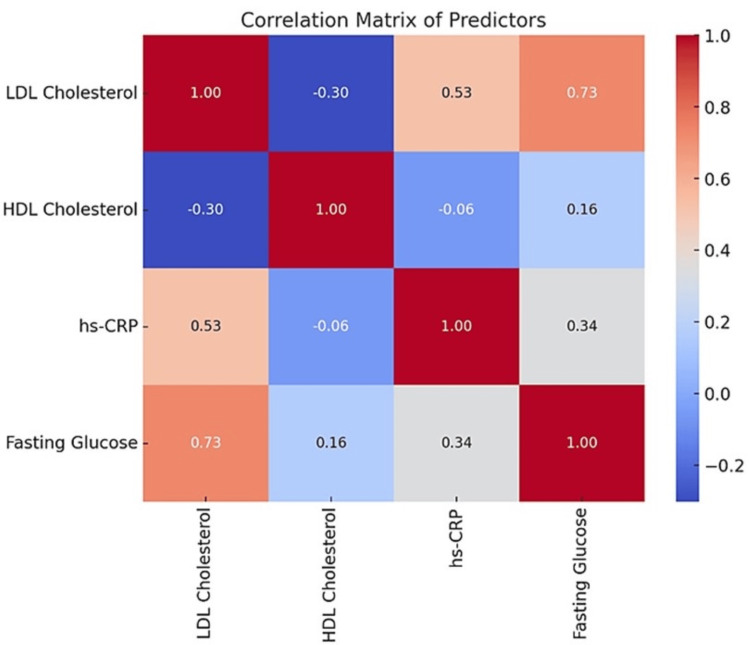
Both LDL Cholesterol and Hs-CRP Show a Moderate Positive Correlation, Indicating That Higher LDL Levels Are Associated With Higher Inflammatory Marker Levels. HDL Cholesterol Has a Weak Inverse Correlation With Hs-CRP, Suggesting That Lower HDL Levels Might Be Linked to Higher Inflammation. Fasting Glucose Shows a Moderate Positive Correlation With Hs-CRP, Reflecting the Role of Hyperglycemia in Systemic Inflammation. LDL: Low-Density Lipoprotein; HDL: High-Density Lipoprotein; hs-CRP: High-Sensitivity C-reactive Protein

The heatmap visually illustrates the correlations among selected key biochemical predictors of CVD, including LDL cholesterol, HDL cholesterol, hs-CRP, and fasting glucose levels. Variables were chosen based on their statistical significance in bivariate analyses and their relevance to the study’s objectives, focusing on independent and non-redundant contributions to CVD risk. The color gradient in the heatmap represents the strength and direction of correlations, with red indicating positive correlations and blue denoting negative correlations. Notably, LDL cholesterol shows a moderate positive correlation with hs-CRP (r ≈ 0.6), highlighting the interplay between elevated LDL levels and increased inflammation. Conversely, HDL cholesterol exhibits a weak negative correlation with hs-CRP (r ≈ -0.3), reflecting its anti-inflammatory properties. Fasting glucose demonstrates a moderate positive correlation with hs-CRP (r ≈ 0.5), emphasizing the role of glucose dysregulation in systemic inflammation (Figure 3).

Interactions between triglycerides, fasting glucose, and other predictors

Triglycerides, fasting glucose, and homocysteine were significant predictors in the initial analyses, demonstrating interactions with other key variables, such as LDL cholesterol and hs-CRP. For instance, triglycerides exhibited a moderate positive correlation with LDL cholesterol (r ≈ 0.5), reflecting their shared role in lipid metabolism and cardiovascular risk. Similarly, fasting glucose correlated moderately with hs-CRP (r ≈ 0.5), highlighting the contribution of glucose dysregulation to systemic inflammation (Figure 2).

Inclusion and exclusion of predictors

Additional potential predictors, including triglycerides, fasting glucose, and homocysteine, were initially included in the regression model. Triglycerides and fasting glucose showed significant associations with CVD in bivariate analyses (triglycerides: F = 10.58, p < 0.001; fasting glucose: F = 11.72, p < 0.001). However, these variables were excluded from the final multivariate regression model due to multicollinearity, as assessed using variance inflation factors (VIF) with a threshold of 10. Homocysteine levels, despite being independently significant (OR = 1.6, 95% CI: 1.3-2.0, p < 0.001), were excluded due to collinearity with hs-CRP. 

Effect size interpretation of odds ratios

The ORs for key predictors were interpreted based on existing literature to assess their effect sizes. Elevated LDL cholesterol (OR = 2.1, 95% CI: 1.7-2.6) represents a moderate to large effect size, aligning with its established role in atherosclerosis. Reduced HDL cholesterol (OR = 1.8, 95% CI: 1.4-2.3) indicates a moderate effect size, underscoring its protective function in reverse cholesterol transport and anti-inflammatory pathways. Elevated hs-CRP (OR = 1.9, 95% CI: 1.5-2.4) also represents a moderate effect size, emphasizing the significance of inflammation in CVD pathogenesis. 

Implications of predictor interrelationships

The heatmap analysis revealed significant correlations among predictors, particularly between LDL cholesterol and hs-CRP (r ≈ 0.6, moderate positive correlation). This relationship highlights the interplay between dyslipidemia and inflammation in promoting atherosclerosis and plaque instability. LDL cholesterol contributes to the formation of oxidized LDL (oxLDL), which exacerbates endothelial damage and triggers an inflammatory response, as evidenced by elevated hs-CRP levels. 

Adjustment for confounders

The regression model accounted for key confounders, including age, gender, smoking, alcohol use, and physical activity, ensuring the robustness of observed associations. For instance, while age was significantly associated with CVD in univariate analyses, its inclusion in the adjusted model did not diminish the independent significance of LDL cholesterol or hs-CRP. This adjustment reinforced the reliability of the predictors.

Multicollinearity and variable exclusion

Multicollinearity among predictors was assessed using VIF values. Variables such as triglycerides and fasting glucose exhibited high VIFs due to their correlation with LDL cholesterol and hs-CRP, respectively, and were excluded to maintain the stability of the model. 

Practical implications of predictors

The practical implications of the identified predictors are significant. Reducing LDL cholesterol by 1 mmol/L is estimated to lower CVD risk by approximately 20% while increasing HDL cholesterol by 1 mmol/L could reduce risk by about 13%. Reducing hs-CRP through lifestyle changes or pharmacological interventions, such as anti-inflammatory therapies, could further mitigate CVD risk. 

Strongest contributors to CVD risk

Among the predictors included in the final regression model, LDL cholesterol (OR = 2.1, 95% CI: 1.7-2.6) and hs-CRP (OR = 1.9, 95% CI: 1.5-2.4) emerged as the strongest contributors to CVD risk, as evidenced by their odds ratios and partial R² values. Low-density lipoprotein cholesterol accounted for the largest proportion of variance in CVD risk (partial R² = 0.18), highlighting its pivotal role in atherosclerosis. hs-CRP, with a partial R² of 0.15, was the second-largest contributor, emphasizing its importance in promoting systemic inflammation and plaque instability. High-density lipoprotein cholesterol (OR = 1.8, 95% CI: 1.4-2.3) also contributed significantly, with a partial R² of 0.12.

Subgroup analyses

Subgroup analyses revealed important demographic variations in predictor significance. For example, LDL cholesterol and hs-CRP were stronger predictors in males, while HDL cholesterol had a greater protective effect in females. Age-stratified analyses indicated that fasting glucose and hs-CRP were more significant in older participants, likely due to the cumulative effects of metabolic and inflammatory changes over time. 

Logistic regression analysis

The logistic regression analysis revealed several significant biochemical predictors of CVD. Elevated LDL cholesterol was associated with a 2.1-fold increased risk of CVD (95% CI: 1.7-2.6, p < 0.001), while low HDL cholesterol levels increased the risk by 1.8 times (95% CI: 1.4-2.3, p < 0.001). Elevated triglycerides also emerged as a significant risk factor, with a 1.5-fold increased likelihood of CVD (95% CI: 1.2-1.9, p=0.002). Hyperglycemia, indicated by fasting blood glucose levels, was associated with a 1.7-fold higher risk (95% CI: 1.4-2.2, p < 0.001). Markers of systemic inflammation, such as hs-CRP, increased the risk of CVD by 1.9 times (95% CI: 1.5-2.4, p < 0.001), while elevated homocysteine levels were associated with a 1.6-fold increased risk (95% CI: 1.3-2.0, p < 0.001) (Table 3). 

**Table 3:** Results of Logistic Regression OR: Odds Ratio; CI: Confidence Interval; LDL: Low-Density Lipoprotein; HDL: High-Density Lipoprotein; hs-CRP: High-Sensitivity C-reactive Protein

Predictor	OR	95% CI for OR	p-value
LDL Cholesterol	2.1	1.7–2.6	<0.001
HDL Cholesterol	1.8	1.4–2.3	<0.001
Triglycerides	1.5	1.2–1.9	0.002
Fasting Blood Glucose	1.7	1.4–2.2	<0.001
hs-CRP	1.9	1.5–2.4	<0.001
Homocysteine	1.6	1.3–2.0	<0.001

Validation of the regression model

The regression model was validated using k-fold cross-validation (k = 5), which confirmed the robustness of the findings and ensured that the results were not specific to the training dataset. The model's overall fit was evaluated using the Hosmer-Lemeshow goodness-of-fit test, which yielded a p-value > 0.05, indicating an adequate fit. Furthermore, the area under the receiver operating characteristic (ROC) curve (AUC) was 0.82, demonstrating good discriminatory power in identifying participants with and without CVD.

## Discussion

This study examined the prevalence and biochemical risk factors for CVD in a South Indian population, highlighting the significant roles of LDL cholesterol, HDL cholesterol, hs-CRP, fasting glucose, and homocysteine as predictors. The findings align with the established understanding of CVD pathogenesis, emphasizing the importance of addressing dyslipidemia, systemic inflammation, and glucose dysregulation.

The study’s CVD prevalence of 273 participants (22.83%) aligns closely with regional reports of 22% to 27% in urban areas and 15% to 18% in rural settings, reflecting the representativeness of the study cohort. Odds ratios for key predictors, including LDL cholesterol (OR = 2.1, 95% CI: 1.7-2.6) and hs-CRP (OR = 1.9, 95% CI: 1.5-2.4), are consistent with findings from large cohort studies [16-17]. These values underscore the critical role of dyslipidemia and inflammation in CVD risk while also highlighting unique contributions from fasting glucose and homocysteine, which reflect the metabolic and nutritional characteristics of the South Indian population.

Elevated LDL cholesterol was identified as a significant predictor of CVD (OR = 2.1, 95% CI: 1.7-2.6, p < 0.001), contributing to atherosclerosis through plaque formation. Oxidized LDL triggers endothelial dysfunction, promoting monocyte recruitment and foam cell formation, which are critical steps in atherogenesis. Conversely, decreased HDL cholesterol levels (428 participants (35.7%), OR = 1.8, 95% CI: 1.4-2.3, p < 0.001) were associated with increased CVD risk. High-density lipoprotein cholesterol plays a protective role by mediating reverse cholesterol transport and exerting anti-inflammatory effects. Similar findings have been reported in the Framingham Heart Study [17], where high LDL and low HDL levels were strongly correlated with CVD events [17-19].

The study identified specific biochemical trends unique to the South Indian cohort, such as elevated hs-CRP levels in 353 participants (29.4%) and elevated homocysteine levels in 223 participants (18.6%). These trends are influenced by dietary patterns dominated by carbohydrate-rich foods (e.g., rice) and low intake of micronutrients such as folate and vitamin B12, which contribute to hyperhomocysteinemia. The observed urban-rural disparity in CVD prevalence (75 participants (27.37%) in urban areas vs. 48 participants (17.51%) in rural areas) highlights the impact of urban-specific risk factors, including sedentary lifestyles, high-calorie diets, and stress. While global findings on homocysteine-lowering therapies have been mixed, localized research is needed to explore the potential of such interventions in the South Indian population. Dietary deficiencies of folate and vitamin B12 are more common in this region, particularly among vegetarians, necessitating targeted interventions like community-based micronutrient supplementation to reduce hyperhomocysteinemia and associated CVD risk. The Justification for the Use of Statins in Primary Prevention (JUPITER) Trial demonstrated that individuals with elevated hs-CRP levels, even in the absence of dyslipidemia, benefitted from statin therapy, which reduced inflammation and subsequent cardiovascular events [20].

Fasting glucose levels were significantly associated with increased CVD risk (mean = 124.6 ± 15.2 mg/dL in participants with CVD vs. 104.8 ± 12.3 mg/dL without CVD, OR = 1.7, 95% CI: 1.4-2.2, p < 0.001). Hyperglycemia contributes to CVD through oxidative stress, endothelial dysfunction, and the formation of advanced glycation end-products (AGEs). Similar associations have been reported in the UK Prospective Diabetes Study (UKPDS), where poor glycemic control was strongly linked to increased cardiovascular events [21]. These findings underscore the need for stringent glycemic management to mitigate CVD risk, particularly in populations with high diabetes prevalence, such as South India.

Elevated homocysteine levels (223 participants (18.6%), OR = 1.6, 95% CI: 1.3-2.0, p < 0.001) were also significantly associated with CVD. Homocysteine induces endothelial injury, promotes oxidative stress, and enhances thrombogenesis, contributing to atherosclerotic progression. Previous studies, such as the Norwegian Vitamin (NORVIT) trial, identified elevated homocysteine as a modifiable risk factor for CVD [22-23]. Despite mixed global findings on homocysteine-lowering therapies, this study highlights its relevance in the Indian context, where dietary deficiencies of folate and vitamin B12 are prevalent.

Regression analysis identified LDL cholesterol, hs-CRP, and HDL cholesterol as the strongest predictors of CVD, with partial R² values of 0.18, 0.15, and 0.12, respectively. These predictors demonstrated consistent significance across validation techniques, including k-fold cross-validation (AUC = 0.82). Interactions between LDL cholesterol and hs-CRP suggest a synergistic effect, with oxLDL contributing to inflammation and exacerbating plaque instability. The exclusion of triglycerides and fasting glucose due to multicollinearity highlights the complexity of interactions among these predictors.

Gender-specific analyses revealed that LDL cholesterol and hs-CRP were stronger predictors in males, while HDL cholesterol had a greater protective effect in females. Age-stratified analyses indicated that fasting glucose and hs-CRP were more significant in older participants, likely due to cumulative metabolic and inflammatory effects. These findings support the need for personalized prevention strategies tailored to gender and age.

These results emphasize the importance of early screening for LDL cholesterol, hs-CRP, and homocysteine, alongside education campaigns promoting dietary changes and physical activity. Emerging therapies, such as IL-6 inhibitors, proprotein convertase subtilisin/kexin type 9 (PCSK9) inhibitors, and glucagon-like peptide-1 (GLP-1) receptor agonists, offer promising options for reducing CVD risk in this population [16-23]. Targeted interventions based on identified risk profiles could significantly mitigate the cardiovascular burden in South India.

Limitations

The study was conducted at a single tertiary care center, which may limit the generalizability of the findings to broader populations, particularly rural and underserved communities. The reliance on self-reported lifestyle data, such as diet and smoking habits, may introduce recall bias, and the inflammatory marker analysis was restricted to hs-CRP, excluding other key biomarkers like IL-6 and TNF-α, which could have provided additional insights into systemic inflammation. Furthermore, the study focused on predictors of CVD rather than long-term outcomes, limiting the ability to assess the temporal impact of these factors over time. Carotid intima-media thickness (CIMT), a valuable measure of subclinical atherosclerosis, was not included due to resource and logistical constraints in conducting imaging studies for a large cohort of 1,200 participants. Instead, the study prioritized widely accessible and scalable biochemical markers to ensure feasibility and applicability in resource-limited settings. These limitations highlight the need for future multicenter, longitudinal studies incorporating advanced biomarker panels and imaging modalities to validate and expand upon these findings.

## Conclusions

This study highlights the significant burden of CVD and its associated risk factors, including elevated inflammatory and metabolic markers. The findings indicate a higher prevalence of CVD in urban populations compared to rural areas, underscoring the role of lifestyle and environmental influences in disease progression. Based on these results, targeted interventions such as dietary guidelines promoting micronutrient-rich foods to address folate and vitamin B12 deficiencies, lipid-lowering therapies, and public health campaigns focusing on physical activity, smoking cessation, and nutrition education may help reduce cardiovascular risk. Additionally, community-based strategies tailored for rural populations are crucial for addressing healthcare disparities and improving early detection and management.
